# Complete genome sequence of *Algoriphagus halophilus* strain SOCE 003, a marine bacterium isolated from the surface seawater of Dapeng Bay

**DOI:** 10.1128/mra.01306-24

**Published:** 2025-01-15

**Authors:** Jiayi Yang, Tian Xia, Xunying Zhou, Shuaishuai Xu, Kangli Guo, Qianqing Zhang, Jing Guo, Shengwei Hou

**Affiliations:** 1 Department of Ocean Science and Engineering, Southern University of Science and Technology 255310 , Shenzhen, China; 2 Department of Ocean Science, Hong Kong University of Science and Technology, Hong Kong, China; 3 Shanghai Key Laboratory of Polar Life and Environment Sciences, Shanghai Jiao Tong University 12474 , Shanghai, China; 4 Key Laboratory of Polar Ecosystem and Climate Change, Ministry of Education, Shanghai Jiao Tong University, Shanghai, China; Montana State University, Bozeman, Montana, USA

**Keywords:** *Algoriphagus*, marine bacterium, genome sequence, nanopore sequencing

## Abstract

*Algoriphagus* is a heterotrophic bacterium commonly found in diverse marine environments. Here, we report the complete genome sequence of *Algoriphagus halophilus* strain SOCE 003, which is 5,154,101 bp long, encoding 5,524 annotated protein-coding genes, 39 tRNAs, and 8 rRNAs. This genome information will help us understand the ecology of *Algoriphagus*.

## ANNOUNCEMENT

The genus *Algoriphagus* represents a group of aerobic heterotrophic bacterium (1). They have been isolated from diverse marine habitats, including seawater (2), cold seeps (3), mangrove rhizosphere (4), and so on. As of November 2024, there are 50 species with valid names (www.bacterio.net/algoriphagus.html) in this genus, and some of them encode metabolic potentials of degrading polysaccharides and other macromolecules (5, 6).

We report the complete genome sequence of *Algoriphagus halophilus* (*A. halophilus*) strain SOCE 003, isolated from the surface seawater of Dapeng Bay (22°52′43″ N, 114°01′93″ E), China. The measured salinity, chlorophyll *a* concentration, and temperature were approximately 30 ppt, 5.8 mg/L, and 24°C, respectively. Seawater was filtered through a 0.22 μm nitrocellulose membrane and incubated on the Marine Broth 2216 agar (BD Difco, NJ, USA) at 28°C for 3 days. Bacterial colonies were isolated using the streak plating method and were cultivated in the 2216 liquid medium to harvest microbial cells. Genomic DNA was extracted using the cetyltrimethylammonium bromide (CTAB) method with proteinase K, followed by chloroform:isoamyl alcohol (24:1) phase separation (7). DNA was precipitated with isopropanol, washed with 70% ethanol, resuspended in TE buffer, and quantified using the Qubit double-stranded DNA broad-range kit (Thermo Fisher Scientific, USA) (7).

Illumina sequencing was performed at Novogene Co., Ltd. (Tianjin, China) on a NovaSeq 6000 platform (Illumina, CA, USA) with the 2 × 150 bp paired-end strategy. Genomic DNA (0.2 μg) was sheared into ~350 bp long fragments using the LE220R-plus ultrasonicator (Covaris, MA, USA), which were then end polished and A-tailed using the NEBNext Ultra DNA library preparation kit (New England Biolabs, MA, USA) following the manufacturer’s instructions. The Illumina sequencing yielded 3.0 Gbps raw reads. Adaptors and low-quality ends were trimmed using fastp v0.19.7 (8) with default parameters, producing 2.6 Gbps clean reads.

**Fig 1:**
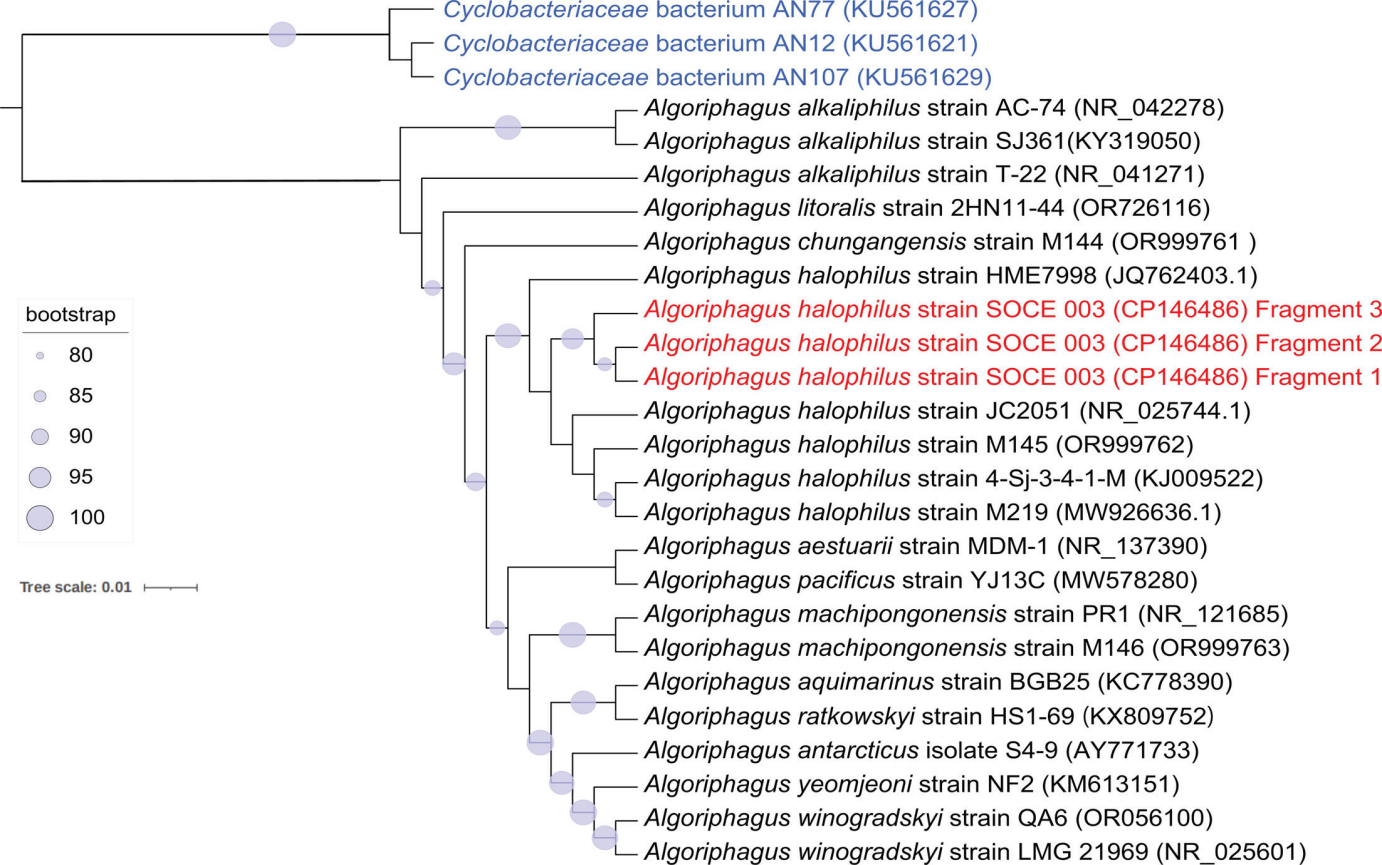
Maximum likelihood phylogenetic tree of *A. halophilus* SOCE 003 with other close relatives. Three 16S rRNA genes were identified in *A. halophilus* SOCE 003 (Fragments 1–3), with the highest pairwise sequence identity of 94.75% (Fragment 1 vs Fragment 2), and the lowest identity of 91.13% (Fragment 2 vs Fragment 3), as calculated using the distmat module of EMBOSS v6.6.0 (17) after alignments using MAFFT v7.453 (18). Public 16S rRNA gene sequences of closely related strains were obtained from the NCBI GenBank database, and only sequences with valid names (www.bacterio.net/algoriphagus.html) were selected. All the 16S sequences were aligned using ClustalW (19), and the maximum likelihood phylogenetic tree was inferred from the alignment using the best-fitting model automatically selected by ModelFinder (20) in IQ-Tree v2.2.0 (16). The tree file was visualized using the iTOL Web server (https://itol.embl.de). The three 16S sequences of *A. halophilus* SOCE 003 are shown in red. Three *Cyclobacteriaceae* bacterium strains (highlighted in blue) were used as the outgroup to root the tree. Branch lengths represent phylogenetic distances from the reference genome. Blue circles represent bootstrap values >80.

DNA fragments >3 kb were selected using the Long Fragment Buffer (Oxford Nanopore Technologies, Oxford, UK), and libraries were prepared with the ligation kit SQK-LSK109 and sequenced on the MinION Mk1C platform with an R9.4.1 flow cell. Raw fast5 files were processed using Guppy v6.1.7 (9) for adapter removal to get clean fastq reads. Nanopore quality control was conducted using NanoPlot v1.40.2 with a *Q*-value cutoff >7 (10). In total, 381,637 Nanopore reads were generated with an *N*_50_ of 6,221 bp and a mean read length of 2,323 bp. An overlap graph was assembled from Nanopore reads using Flye v2.9 (11), and overlaps between contigs were trimmed with default parameters. Unicycler v0.5.1 (12) was used to assemble Nanopore contigs with Illumina clean reads, and the circular contig was identified and rotated to begin with the *dnaA* gene on the forward strand. This assembly was corrected using Pilon v1.24 (13) and NextPolish v1.4.0 (14) with Illumina reads, resulting in a complete genome of 5,154,101 bp in size, with a GC content of 39%. All tools used default parameters unless noted. Genome annotation was done using the NCBI Prokaryotic Genome Annotation Pipeline (PGAP) (15), which predicted 5,524 protein-coding genes, 39 tRNAs, and 8 rRNAs (5S, 16S, and 23S). A maximum likelihood phylogenetic tree was constructed based on 16S rRNA genes using IQ-Tree v2.2.0 (16) (Fig. 1). The first 16S rRNA gene (Fragment 1) of *A. halophilus* SOCE 003 strain showed the highest identity (91.15%) to that of *A. halophilus* JC2051 (NR_025744.1). The complete genome sequence of *A. halophilus* SOCE 003 serves as a reference to decipher the metabolism and adaptation of *Algoriphagus* in diverse marine habitats.

## Data Availability

The complete genome sequence of *Algoriphagus halophilus* strain SOCE 003 has been deposited at GenBank under the BioProject accession number PRJNA1082223, the BioSample accession number SAMN40200481, and the GenBank accession number CP146486. The SRA accession numbers of the Illumina and Nanopore reads are SRR30551005 and SRR30565972, respectively.
